# Non-AIDS Associated Kaposi's Sarcoma: Clinical Features and Treatment Outcome

**DOI:** 10.1371/journal.pone.0018397

**Published:** 2011-04-12

**Authors:** Lena Jakob, Gisela Metzler, Ko-Ming Chen, Claus Garbe

**Affiliations:** Department of Dermatology, Eberhard-Karls-University, Tuebingen, Germany; The University of Queensland, Australia

## Abstract

**Background:**

Kaposi's sarcoma (KS) in HIV negative patients is rare and has to be distinguished from AIDS associated KS. Two groups are at risk to develop non-AIDS related KS: elderly men mainly of Mediterranean origin and persons with iatrogenic immunosuppression.

**Patients and Methods:**

In order to define risk-groups and major clinical features we retrospectively evaluated clinical data of all patients with non-AIDS associated KS presenting to the Department of Dermatology, University Hospital Tuebingen between 1987 and 2009. Data were extracted from the tumor registry of the Comprehensive Cancer Center Tuebingen and from patient records.

**Results:**

20 patients with non-AIDS KS have been identified. The average age at KS onset was 66.6 years; the male-to-female-ratio was 3∶1. Most of the patients were immigrants from Mediterranean or Eastern European countries (60%). 15 cases of classic KS versus 5 cases of iatrogenic KS were observed. In 95% of the cases, KS was limited to the skin, without mucosal, lymph node or visceral manifestation. KS lesions were in all cases multiple and mostly bilateral, the most common localization was the skin of the lower extremities. Tumor control was achieved in nearly all cases by the use of local or systemic therapy. No patient died from KS.

**Conclusions:**

Unlike KS in AIDS patients, non-AIDS associated KS is a rather localized process which rarely involves lymph nodes or organs. It is mostly seen in elderly males from Mediterranean or Eastern European countries and in most cases responsive on local or systemic therapeutic strategies.

## Introduction

Kaposi's sarcoma (KS) was first described in 1872 by the Hungarian dermatologist Moritz Kaposi [1]. It is a rare neoplasm of lymphatic endothelial cells frequently evident as multiple vascular cutaneous and mucosal nodules. Lymph node and visceral manifestation is seen in cases of strong immunosuppression or aggressive disease.

Four groups are at risk to develop KS: elderly males of Mediterranean and Eastern European lineage; children and adults from central Africa; persons who are iatrogenically immunocomromised; and homosexual men infected with human immunodeficiency virus (HIV) [2]. Major differences in clinical presentation and in prognosis among those groups have lead to the following classifications: classical KS, endemic or so called African KS, iatrogenic KS and AIDS-KS.

Chang et al. discovered human herpes virus 8, (HHV-8), also known as Kaposi's sarcoma associated herpes virus, which is strongly implicated in the pathogenesis of all types of KS [3]. The herpes virus is considered necessary but not sufficient for the development of KS, which is a multistep process including not only HHV-8 infection, but also genetic and angiogenic factors, as well as the production of several inflammatory cytokines [4].

To date it remains unclear whether KS itself is a true malignancy or rather just a reactive proliferation [5].

This study focuses on non-AIDS related KS of the skin i.e. cutaneous manifestations of KS in HIV-negative patients presented to the Department of Dermatology, University of Tuebingen Medical Center between 1987 and 2009.

## Materials and Methods

This is a retrospective descriptive study of a series of 20 patients admitted consecutively for KS, from 1987 to 2009 in the Department of Dermatology, University of Tuebingen Medical Center. Informed consent was not obtained for all patients as the entire data was analyzed anonymously. This applies according to the German Medical Association's professional code of conduct and has been approved by the Ethics Committee, University of Tuebingen (Figure S1).

In order to define risk-groups and major clinical features we retrospectively identified cases registered by the tumor registry of the Comprehensive Cancer Center Tuebingen and evaluated clinical data documented in the hospital records.

All cases presented histopathologically approved KS lesions of the skin and negative HIV-1/2 screening by macro enzyme immunoassay. Histological diagnosis was in most cases completed by immunohistochemical tests such as HHV-8 staining, or immunostaining with antibodies against endothelial markers D2-40, CD31 and CD34. Most patients underwent tumor staging by lymph node and abdominal ultrasound as well as chest x-ray.

Demographic features such as origin, age at onset, gender of the patient, as well as clinical features such as clinical subtype of KS and localization of lesions were evaluated. Furthermore treatment modalities, results and tumor recurrence in the time of observation were recorded. Treatment outcome was classified according to the Response Evaluation Criteria in Solid Tumors (RECIST Guidelines) [6]. One patient had a stable disease course and therefore did not need treatment and one patient refused therapy. Median time of follow-up for all patients were four years (*SEM* = 7.86 months). In 7 cases time of observation was determined by intercurrent death. Causes of death were other than KS in all cases.

Statistical package of social sciences 16.0 software (SPSS Inc., Chicago, IL, U.S.A.) was used to calculate means and standard deviations.

## Results

20 cases of non-AIDS KS were identified in this study. Mean age at diagnosis of the group was 66.6 year-old (*SD* = 15.36). The youngest patient was 36-year-old and iatrogenically immunosuppressed; the oldest developed his classic KS by the age of 90.

Mean age at onset of patients with classic KS was 69.6 years (*SD* = 12.09).

A male predominance in KS – 75% male patients (*n* = 15) versus 25% female patients (*n* = 5) was shown with a male/female ratio of 3∶1.

60% (*n* = 12) of the patients observed in Tuebingen were immigrants from Mediterranean and Eastern European countries (all first generation) versus eight German patients.

Regarding the clinical Subtype 15 cases of classic KS and five cases of iatrogenic KS were identified.

Iatrogenic immunosuppression was in one case used for a liver transplant-patient and four patients were under immunosuppressive therapy due to autoimmune diseases i.e. Behçet's disease, myasthenia gravis, chronic membranous glomerulonephritis and systemic sarcoidosis. Immunosuppressive medication included systemic corticosteroids in all five cases in addition to azathioprine in one case and ciclosporin A in two cases.

One patient was categorized as classic KS even though presenting a primary nodal peripheral T-cell lymphoma causing immunosuppression. However, immunosuppression was in this case not iatrogenically induced.

In 95% (*n* = 19) of the cases, KS was limited to the skin, without mucosal, lymph node or visceral manifestation.

One patient presented mucosal lesions of the oral and genital region as well as inguinal lymph node invasion.

KS lesions were multiple in all patients (*n* = 20), no patient presented just one singular lesion. 70% of the patients (*n* = 14) were affected on both hemispheres of the body and 30% (*n* = 6) presented unilateral lesions.

The most frequent manifestation was the skin of the lower leg (*n* = 8), six patients were affected on their feet and five patients on all four extremities. All in all 95% of the patients were affected on the skin of the lower extremities.

**Figure 1:**
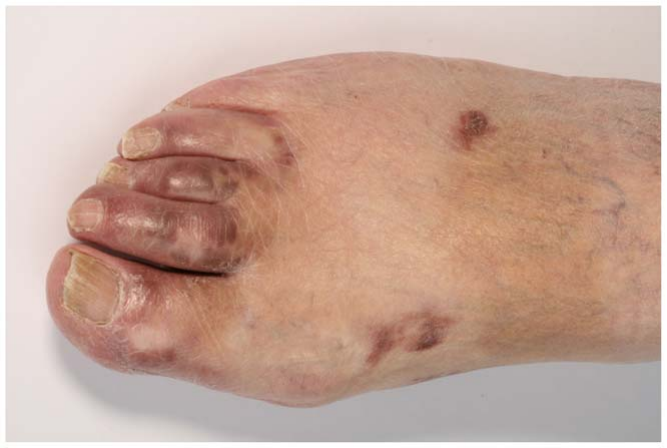
Brownish macules and plaques on the foot of a patient with classic KS.

**Figure 2:**
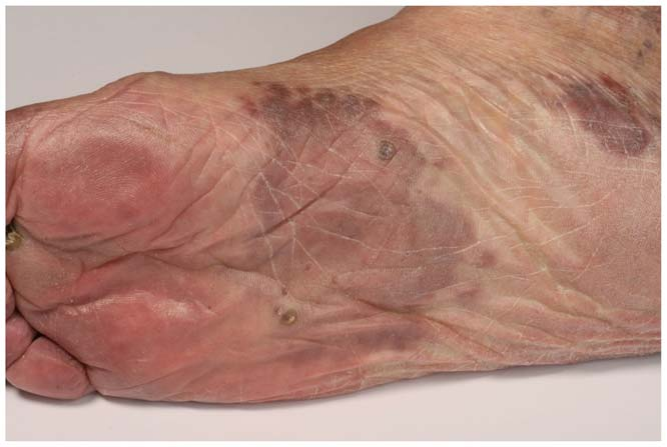
Patch stage classic KS: Red to brownish irregularly-shaped macules and plaques.

Figure 1 and 2 present a patch stage classic KS characterized by several brownish irregularly-shaped maculae as well as the existence of a few partly indurated plaques.

**Figure 3:**
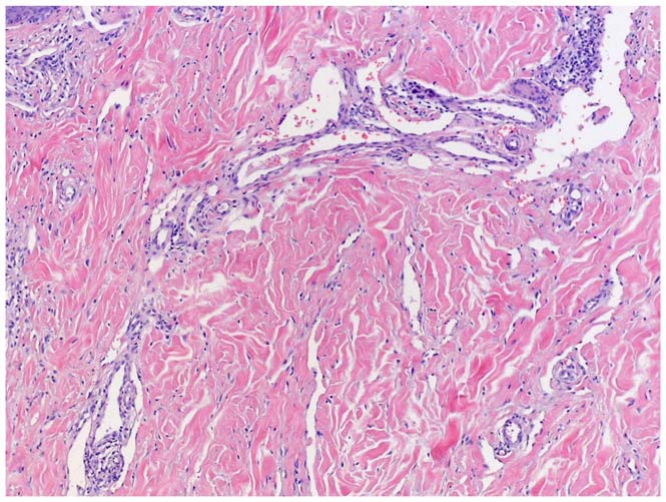
Patch stage KS with promontory sign. Dilated irregular vascular channels surround a pre-existing vessel.

All stages of KS lesions were observed within the group: The initial patch stage exhibits an irregular proliferation of jagged vascular channels in the dermis below an integral epidermis. The so-called promontory sign is sometimes found in patch stage lesions and denotes vascular spaces surrounding pre-existing blood vessels (Figure 3). Perivascular lymphoplasmocytic cells as well as extravasated erythrocytes and hemosiderin deposits are characteristic for patch stage.

**Figure 4:**
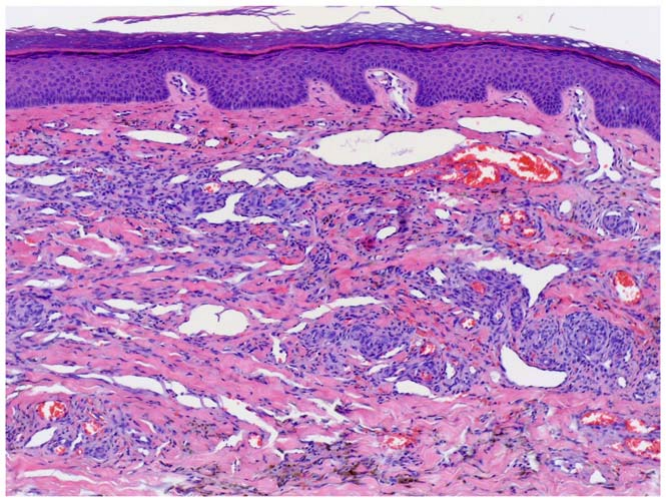
Plaque stage KS with bizarre vessels dissecting the upper dermis. There is erythrocyte extravasation and hemosiderin pigmentation.

**Figure 5:**
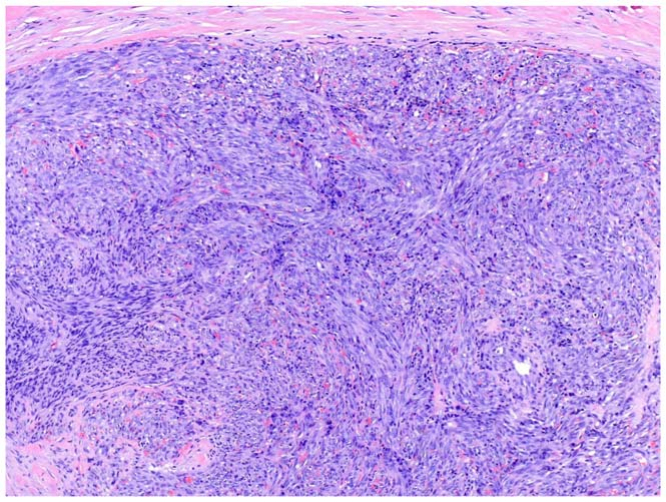
Tumor stage KS: Well-circumscript spindle-cell tumor. Erythrocytes lie within poorly defined slit-like vascular spaces.

**Figure 6:**
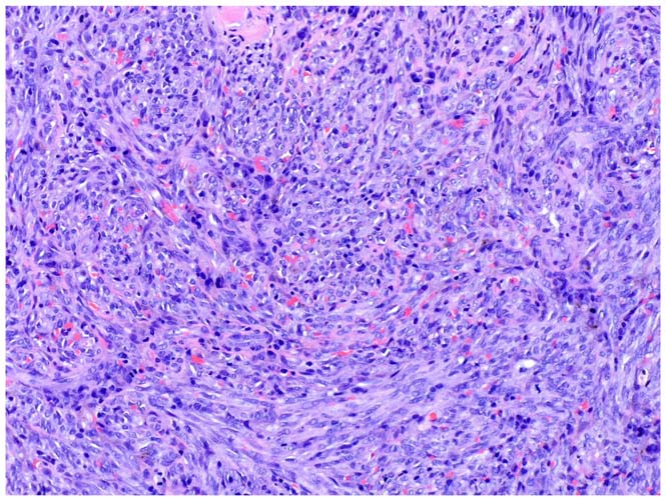
Tumor stage KS: Close up view.

As KS progresses to the plaque stage bizarre vessels with an atypical endothelial lining dissect the collagen tissue (Figure 4). In the nodular stage spindled endothelial cells predominate forming slit-like vascular spaces containing erythrocytes and hyaline globules. Figure 5 and 6 demonstrate a well-circumscript spindle-cell tumor with poorly defined slit-like vascular spaces.

**Figure 7:**
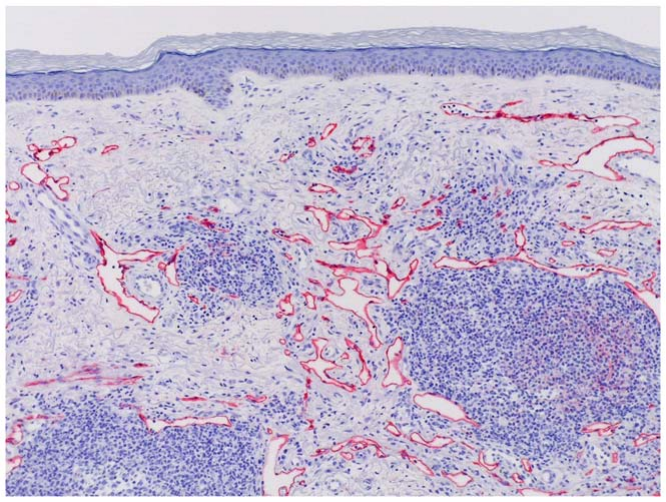
D2-40 staining of arborizing vascular structures; irregular lymphatic vessels.

**Figure 8:**
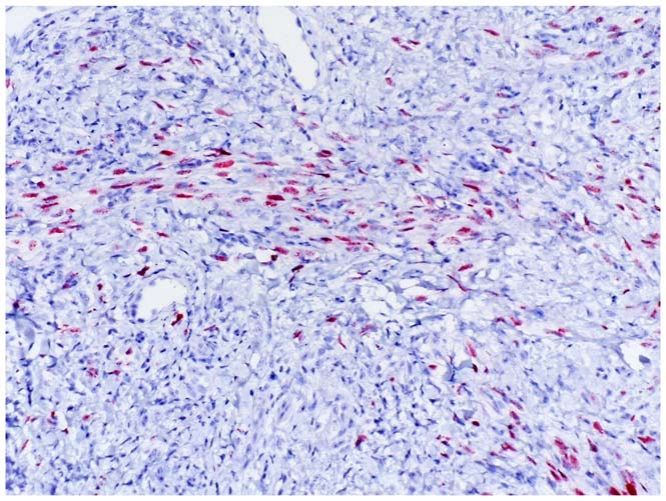
HHV-8 staining of atypical endothelial cells.

Figure 7 shows D2-40 immune staining of lymphatic endothelial cells revealing arborizing vascular structures and lymphocytic cell infiltration. Figure 8 displays HHV-8 stained atypical endothelial cells.

Treatment modalities included local therapy such as surgery and radiotherapy as well as systemic immune therapy with interferon-α-2a. Interferon-α-2a was applied by subcutaneous injection using 3 million international units three times weekly. One patient developed neutralizing antibodies and therefore his therapy was switched to Interferon alphacon (consensus interferon 9 µg daily, then every second day). The duration of administration of interferon ranged from five weeks to six years. In one case with post-transplant KS immunosuppressive medication was switched from ciclosporin A to sirolimus resulting in a partial response. One patient had a stable disease course and therefore did not need treatment and one patient refused therapy.

Treatment outcome was evaluated according to the Response Evaluation Criteria in Solid Tumors (RECIST-Guidelines). The term “complete response” means clearance of KS lesions on later visits when compared with the first lesions on admission. “Partial response” equals at least 30 percent decrease in the sum of the longest diameter of target lesions whereas “stable disease” involves less than 30 percent decrease. The terms complete and partial response as well as stable disease implicate the absence of new lesions or of progressive lesions. This was achieved in all cases. No patient presented a progressive disease course initially nor died of KS. Four patients developed multiple local recurrences. Two of them had primarily received a local therapy and one interferon-α-2a.

Clinical features and treatment outcome of non-AIDS KS are presented in Table 1.

**Table 1:** Clinical features and treatment outcome.

Patient	Age	Origin	Subtype^a^	Localization^b^	Treatment^c^	Result^d^	Recurrence
P1	44	Italy	immune	lower legs	sirolimus	PR	-
P2	87	Germany	classic	lower legs	IFNα	SD	-
P3	76	Romania	classic	lower legs	Rx, IFNα	SD	-
P4	70	Germany	immune	lower legs	Rx, IFNα	CR	-
P5	59	Turkey	classic	extremities	excs.	CR	multiple
P6	69	Italy	classic	lower leg	*No need*	-	-
P7	48	Egypt	immune	lower legs	excs.	CR	multiple
P8	78	Germany	classic	extremities, mucosa, LN	Rx, excs.	SD	multiple
P9	51	Turkey	classic	foot	excs.	CR	-
P10	45	Italy	classic	foot	IFNα	PR	-
P11	89	Germany	classic	feet	Rx, excs.	SD	-
P12	36	Turkey	immune	extremities	IFNα	CR	-
P13	69	Italy	classic	extremities	*drop-out*	-	-
P14	71	Germany	classic	extremities	IFNα	SD	-
P15	70	Italy	immune	feet	Rx	PR	-
P16	68	Germany	classic	feet	IFNα	CR	multiple
P17	62	Turkey	classic	feet	IFNα	CR	-
P18	70	Italy	classic	lower leg	IFNα, excs.	CR	-
P19	79	Germany	classic	nose	excs.	CR	-
P20	90	Germany	classic	lower legs	Rx	CR	-

*Annotations:*

a clinical subtype: immune = iatrogenic immunosuppression;

b main localization: LN = lymph nodes;

c treatment: IFN = interferon-α-2a, Rx = radiotherapy, excs. = excision;

d result: PR = partial response, CR = complete response, SD = stable disease.

## Discussion

Non-AIDS KS is considered a rare disease, but incidence varies according to individual factors such as origin, sex, age and immune status of the patient.

Between 1987 and 2009, only 20 cases of non-AIDS related KS were observed at the Department of Dermatology in Tuebingen, Germany. This is in line with the low incidence of cases appearing in North-Western Europe.

No data regarding the incidence of KS in Germany currently exists. Iscovich et al. analyzed reports of classic KS from different cancer registries throughout the world between 1988 and 1992. Low incidence rates were found in Denmark (0.18 per million men aged over 65 per year), intermediate rates in France, and especially high rates in Sardinia and Sicily (13.2 per million men aged over 65 per year) [7].

It is worth mentioning that the majority of the non-AIDS KS in Tuebingen (12 out of 20) have been found in first generation immigrants from Mediterranean countries with presumably higher incidence rates e.g. in Turkey and Italy (1 per 100,000 men per year [8]). Immigrants from those two countries also represent the two largest ethnic minorities in Germany.

Mean age at onset of our 20 patients was 66.6 years. Focusing exclusively on classic KS an average age of 69.6 years was found, which is comparable to more comprehensive investigations like the Italian 870-cases study conducted by Dal Maso et al. showing a mean age of 72 years [8].

Gender presents another factor which strongly influences KS manifestation. Former male-to-female ratios from 10∶1 to 15∶1 had been reported in classic KS [9]. More recent studies showed gender ratios ranging from 1∶1 in England up to 4∶1 in Italy [10], [11]. Iscovich and colleagues ascertained a lower gender difference in populations with lower incidence rates [7]. The Tuebingen case group showed a significant male predominance with a male-to-female ratio of 3∶1.

Just as other investigations classic KS was the most frequent subtype we found. Iatrogenic KS was with 25% a considerately highly represented subtype. This may be an effect of mismatch in classification. Other studies call this subtype post transplant-KS and patients with KS under immunosuppressive therapy for autoimmune disorders may not be listed in this group. However the increased risk of KS in iatrogenically immunosuppressed patients is well documented [12]–[14].

One of our patients presented with a nodal peripheral T-cell lymphoma, a hematological malignancy that possibly implicates immunosuppression. Over fourfold significant increase of Kaposi's sarcoma in patients with lymphohematopoietic malignancies have been reported in the literature [15]. Presumably immunodeficiency of any kind; iatrogenic, malignant or HIV-induced is a considerable factor in the development of KS.

Clinically, non-AIDS KS mostly presents itself as multiple bilateral cutaneous lesions of the lower limb [7]. We found the lesions to be multiple in 100% of the cases, they were mostly bilateral (70%) and the lower extremity was clearly the most affected localization (95%). Only one patient with extracutaneous lesions was identified. This is in line with the results of Hong and Lee who compared characteristics of KS in HIV positive and negative subjects [16].

KS can be seen as a systemic disease with mutilocular occurrence of vascular tumors. Thus the therapeutic administration of KS differs essentially from the management of most other neoplastic diseases. In comparison with other tumors KS therapy comprises growth control rather than elimination without presenting a palliative situation. A standard therapeutic guideline does not exist as the therapeutic options have to be chosen depending on subtype and stage of the disease as well as on the immune status of the patient [17].

Treatment modalities comprise local therapy for example surgery, radiotherapy and local chemotherapy such as injections of vinca alkaloids or local immune therapy by interferon,9 cis retinoid acid or imiquimod [18]–[23]. Patients with widespread disease may need systemic chemotherapeutic or immunologic medication. Positive results have been found for pegylated liposomal doxorubicin, danaurubicin, paclitaxel and interferon α [24], [25]. In patients with iatrogenic KS, immunosuppressive medication may be reduced or modified with the considerate possibility of grafts being rejected with insufficient immunosuppression [26].

Between 1987 and 2009 single KS lesions have been surgically removed and irradiated. The immune stimulating, antiviral and anti-proliferative properties of interferon-α-2a have been used for both systemic and intralesional therapy. In one patient with post-transplant KS immunosuppressive medication was switched from ciclosporin to the m-TOR inhibitor sirolimus. Sirolimus has immunosuppressive, anti-angiogenic and anti-neoplastic potential [27]. Current studies confirm it's positive effects on iatrogenic KS [28].

We achieved complete responses in half of the patients. The rest presented partial remissions or stable disease courses. No patient died of KS. Due to the small number of cases we were not able to demonstrate differences between treatments, neither were we able to provide outcome diagnosis between different subtypes. Four patients developed multiple local recurrences. Three of them had primarily received a surgical therapy and one had primarily been treated with interferon-α-2a.

In summary this study analyzed the clinical manifestation, treatment and outcome of all patients with non-AIDS associated KS patients presenting to the Department of Dermatology, University Hospital Tuebingen, between 1987 and 2009. In addition to HHV-8 infection, individual factors like origin, age, sex and immune status of the patient seem to have an impact on the development of KS. In contrast to AIDS-associated KS, KS in HIV negative patients appears less aggressive, mostly limited to the skin and well-responsive on local or systemic therapeutic strategies.

## Supporting Information

Figure S1 Ethical vote for the study.
(TIF)

## References

[1] Kaposi M (1872). Idiopathisches multiples Pigmentsarkom der Haut.. Arch Dermatol Syph.

[2] Antman K, Chang Y (2000). Kaposi's sarcoma.. N Engl J Med.

[3] Chang Y, Moore PS (1996). Kaposi's Sarcoma (KS)-associated herpesvirus and its role in KS.. Infect Agents Dis.

[4] Ensoli B, Sgadari C, Barillari G, Sirianni MC, Sturzl M (2001). Biology of Kaposi's sarcoma.. Eur J Cancer.

[5] Goh SG, Calonje E (2008). Cutaneous vascular tumours: an update.. Histopathology.

[6] Therasse P, Arbuck SG, Eisenhauer EA, Wanders J, Kaplan RS (2000). New guidelines to evaluate the response to treatment in solid tumors. European Organization for Research and Treatment of Cancer, National Cancer Institute of the United States, National Cancer Institute of Canada.. J Natl Cancer Inst.

[7] Iscovich J, Boffetta P, Franceschi S, Azizi E, Sarid R (2000). Classic kaposi sarcoma: epidemiology and risk factors.. Cancer.

[8] Dal ML, Polesel J, Ascoli V, Zambon P, Budroni M (2005). Classic Kaposi's sarcoma in Italy, 1985–1998.. Br J Cancer.

[9] Ronchese F, Kern AB (1953). Kaposi's sarcoma (angioreticulomatosis).. Postgrad Med.

[10] Grulich AE, Beral V, Swerdlow AJ (1992). Kaposi's sarcoma in England and Wales before the AIDS epidemic.. Br J Cancer.

[11] Geddes M, Franceschi S, Barchielli A, Falcini F, Carli S (1994). Kaposi's sarcoma in Italy before and after the AIDS epidemic.. Br J Cancer.

[12] Hoshaw RA, Schwartz RA (1980). Kaposi's sarcoma after immunosuppressive therapy with prednisone.. Arch Dermatol.

[13] Klepp O, Dahl O, Stenwig JT (1978). Association of Kaposi's sarcoma and prior immunosuppressive therapy: a 5-year material of Kaposi's sarcoma in Norway.. Cancer.

[14] Penn I (1979). Kaposi's sarcoma in organ transplant recipients: report of 20 cases.. Transplantation.

[15] Royle JS, Baade P, Joske D, Fritschi L (2010). Risk of second cancer after lymphohematopoietic neoplasm.. Int J Cancer.

[16] Hong A, Lee CS (2002). Kaposi's sarcoma: clinico-pathological analysis of human immunodeficiency virus (HIV) and non-HIV associated cases.. Pathol Oncol Res.

[17] Vogt T, Brockmeyer N, Kutzner H, Schofer H (2008). Short German guidelines: angiosarcoma and Kaposi sarcoma.. J Dtsch Dermatol Ges.

[18] Trakatelli M, Katsanos G, Ulrich C, Kalabalikis D, Sotiriadis D (2010). Efforts to counteract locally the effects of systemic immunosupression: a review on the use of imiquimod, a topical immunostimulator in organ transplant recipients.. Int J Immunopathol Pharmacol.

[19] Celestin Schartz NE, Chevret S, Paz C, Kerob D, Verola O (2008). Imiquimod 5% cream for treatment of HIV-negative Kaposi's sarcoma skin lesions: A phase I to II, open-label trial in 17 patients.. J Am Acad Dermatol.

[20] FDA (1998). KS drug goes to FDA. Food and Drug Administration.. GMHC Treat Issues.

[21] Schwartz RA, Micali G, Nasca MR, Scuderi L (2008). Kaposi sarcoma: a continuing conundrum.. J Am Acad Dermatol.

[22] Szajerka T, Jablecki J (2007). Kaposi's sarcoma revisited.. AIDS Rev.

[23] Brambilla L, Bellinvia M, Tourlaki A, Scoppio B, Gaiani F (2010). Intralesional vincristine as first-line therapy for nodular lesions in classic Kaposi sarcoma: a prospective study in 151 patients.. Br J Dermatol.

[24] Di LG, Kreuter A, Di TR, Guarini A, Romano C (2008). Activity and safety of pegylated liposomal doxorubicin as first-line therapy in the treatment of non-visceral classic Kaposi's sarcoma: a multicenter study.. J Invest Dermatol.

[25] Brambilla L, Romanelli A, Bellinvia M, Ferrucci S, Vinci M (2008). Weekly paclitaxel for advanced aggressive classic Kaposi sarcoma: experience in 17 cases.. Br J Dermatol.

[26] Montagnino G, Bencini PL, Tarantino A, Caputo R, Ponticelli C (1994). Clinical features and course of Kaposi's sarcoma in kidney transplant patients: report of 13 cases.. Am J Nephrol.

[27] Sehgal SN (2003). Sirolimus: its discovery, biological properties, and mechanism of action.. Transplant Proc.

[28] Stallone G, Schena A, Infante B, Di PS, Loverre A (2005). Sirolimus for Kaposi's sarcoma in renal-transplant recipients.. N Engl J Med.

