# Supplementary figures and images for: Non-AIDS Associated Kaposi's Sarcoma: Clinical Features and Treatment Outcome
Source: PLoS One. 2011 Apr 12;6(4):e18397. doi: 10.1371/journal.pone.0018397 (PMC3075253; doi:10.1371/journal.pone.0018397)

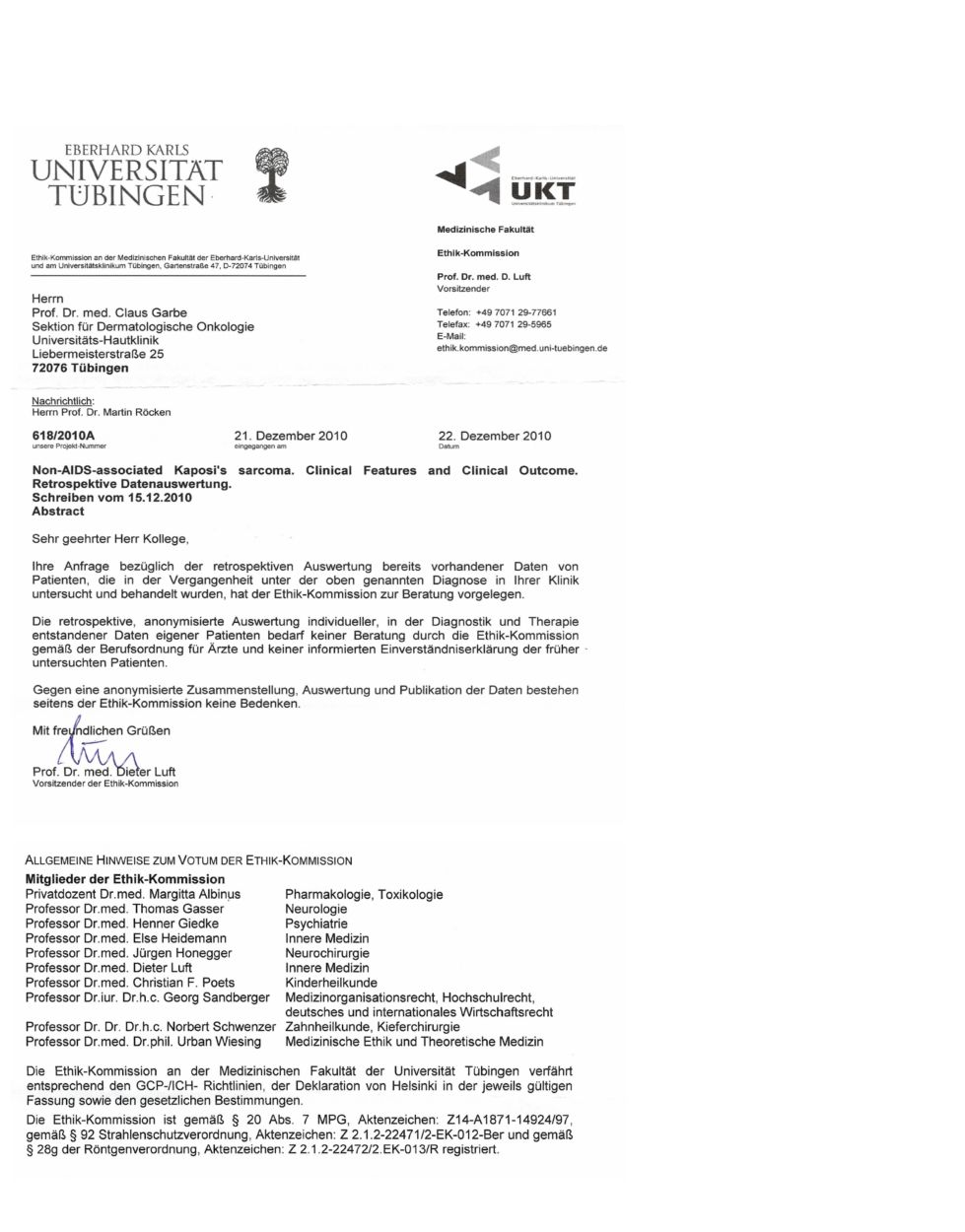

Supplement: Figure S1 — Ethical vote for the study. (TIF) [file pone.0018397.s001.tif]
